# Predicting protein-protein interactions in *Arabidopsis thaliana *through integration of orthology, gene ontology and co-expression

**DOI:** 10.1186/1471-2164-10-288

**Published:** 2009-06-29

**Authors:** Stefanie De Bodt, Sebastian Proost, Klaas Vandepoele, Pierre Rouzé, Yves Van de Peer

**Affiliations:** 1 Department of Plant Systems Biology, Flanders Interuniversity Institute for Biotechnology (VIB), Technologiepark 927, B-9052 Gent, Belgium; 2 Department of Plant Biotechnology and Genetics, Gent University, Technologiepark 927, B-9052 Gent, Belgium

## Abstract

**Background:**

Large-scale identification of the interrelationships between different components of the cell, such as the interactions between proteins, has recently gained great interest. However, unraveling large-scale protein-protein interaction maps is laborious and expensive. Moreover, assessing the reliability of the interactions can be cumbersome.

**Results:**

In this study, we have developed a computational method that exploits the existing knowledge on protein-protein interactions in diverse species through orthologous relations on the one hand, and functional association data on the other hand to predict and filter protein-protein interactions in *Arabidopsis thaliana*. A highly reliable set of protein-protein interactions is predicted through this integrative approach making use of existing protein-protein interaction data from yeast, human, *C. elegans *and *D. melanogaster*. Localization, biological process, and co-expression data are used as powerful indicators for protein-protein interactions. The functional repertoire of the identified interactome reveals interactions between proteins functioning in well-conserved as well as plant-specific biological processes. We observe that although common mechanisms (e.g. actin polymerization) and components (e.g. ARPs, actin-related proteins) exist between different lineages, they are active in specific processes such as growth, cancer metastasis and trichome development in yeast, human and Arabidopsis, respectively.

**Conclusion:**

We conclude that the integration of orthology with functional association data is adequate to predict protein-protein interactions. Through this approach, a high number of novel protein-protein interactions with diverse biological roles is discovered. Overall, we have predicted a reliable set of protein-protein interactions suitable for further computational as well as experimental analyses.

## Background

The complex regulation of diverse biological processes acting in eukaryotic organisms is only possible through interactions between different components in the cell. Proteins, for instance, can be part of extensive complexes, such as transcription factor complexes for the combinatorial control of their target genes or proteasome complexes for protein degradation. It is the specific timing and location of the activity of these protein complexes that defines their role in the cell. Several attempts have been made to infer protein-protein interaction maps in diverse model organisms through large-scale experimental methods [1,2]. In yeast [3-8], human [9,10], Drosophila [11] and C. elegans [12], genome-wide Y2H screens and large-scale affinity purification/mass spectrometry studies have been performed. Nevertheless, the reliability of the results of these studies is thought to be relatively poor because in general quite a small overlap between datasets of experimentally identified interactions is observed. However, there is growing evidence that this observation is due to the complementarity of different methods (e.g. sensitivity of Y2H versus TAP, or different experimental conditions) rather than to a high number of false interactions [8]. Yu et al. (2008) conclude that both Y2H and affinity-purification followed by mass spectrometry (AP/MS) data are of equally high quality but of a fundamentally different and complementary nature. These authors show that, compared to interaction maps based on complex purification and identification, the binary interaction map of yeast proteins is enriched for transient signaling interactions and inter-complex connections [8]. In any case, assessment of the data quality is necessary, not only to design future experiments but also to construct high confidence datasets (or gold standard datasets) used for the training and evaluation of computational methods [13-16]. Several efforts have been made to centralize protein-protein interaction data through the construction of databases such as DIP [17], MINT [18], BioGRID [19] and IntAct [20].

To make full use of the currently available interaction data, computational methods are being developed to assess the quality of experimentally generated protein-protein interactions and to predict new interactions [2,21,22]. Whereas earlier analyses focused on the relation between gene expression and protein-protein interaction only [12,23-26], the integration of several lines of evidence (further referred to as genomic features) in the prediction or validation of protein-protein interactions is highly valued in recent studies, as it increases the performance as well as the coverage of the method [15,27-29]. Typically used genomic features encompass (1) functional features such as Gene Ontology (GO) annotation of the proteins, co-expression of the encoding genes, coordinated protein abundance and co-essentiality, (2) structural features such as co-occurrence of protein domains and overrepresented sequence motifs, (3) comparative genomics-based features such as orthology and, primarily exploited in prokaryotes, phylogenetic profiles, gene neighborhood, co-evolution, and Rosetta Stone (gene split or fusion), and (4) network topology-based features, such as connectivity [30,31,21,27]. In principle, two approaches can be discerned in the prediction of protein-protein interactions. The first approach starts from protein pairs that are identified to be orthologous to known interacting proteins in other species (interolog detection). The interolog detection strategy was initially developed to transfer information on protein-protein interactions from yeast to higher organisms [32-34]. This method assumes that protein-protein interactions are conserved between organisms and that pairs of proteins whose orthologs are known to interact in other species probably interact in the species of interest as well. Although some shortcomings can be identified, protein complexes do show evolutionary conservation [35,36]. Numerous studies have been published in which, mainly human protein-protein interactions are predicted based on interolog detection [37,38]. Furthermore, predictions are made through integrative approaches in a probabilistic framework [39-41]. Other studies start from all possible protein pairs, often incorporating interolog detection as a genomic feature or do not include interolog detection at all [27,42]. The latter approach has the advantage that interactions do not need to be conserved over long evolutionary distances, but often identify associations between genes rather than protein-protein interactions [28,29,43,44].

For the model plant *Arabidopsis thaliana*, attempts to construct large-scale protein-protein interaction maps as well as the application and critical assessment of computational methods have been rather limited [45,46]. In this study, we aim to predict a reliable set of protein-protein interactions suitable for experimental validation as well as further computational analyses. First of all, we investigate whether the necessary assumptions taken in our approach are valid in the model plant *Arabidopsis thaliana*: namely, (1) (some) protein-protein interactions in yeast and animals (source organisms) are conserved in *Arabidopsis thaliana *(target organism), (2) interacting proteins co-localize, (3) interacting proteins function in the same biological process, and (4) genes encoding interacting proteins show similar expression patterns. Hereby, the relative contribution of these features to the prediction of protein-protein interactions in *Arabidopsis thaliana *is assessed. The prediction of Arabidopsis protein-protein interactions is performed, exploiting the conservation of these interactions between species on the one hand, and utilizing functional association data on the other hand. Finally, protein complexes are delineated from the predicted protein-protein interaction network and the function and evolutionary conservation of these protein complexes is studied.

## Results

### Integration of orthology, GO annotation and gene expression

**Figure 1:**
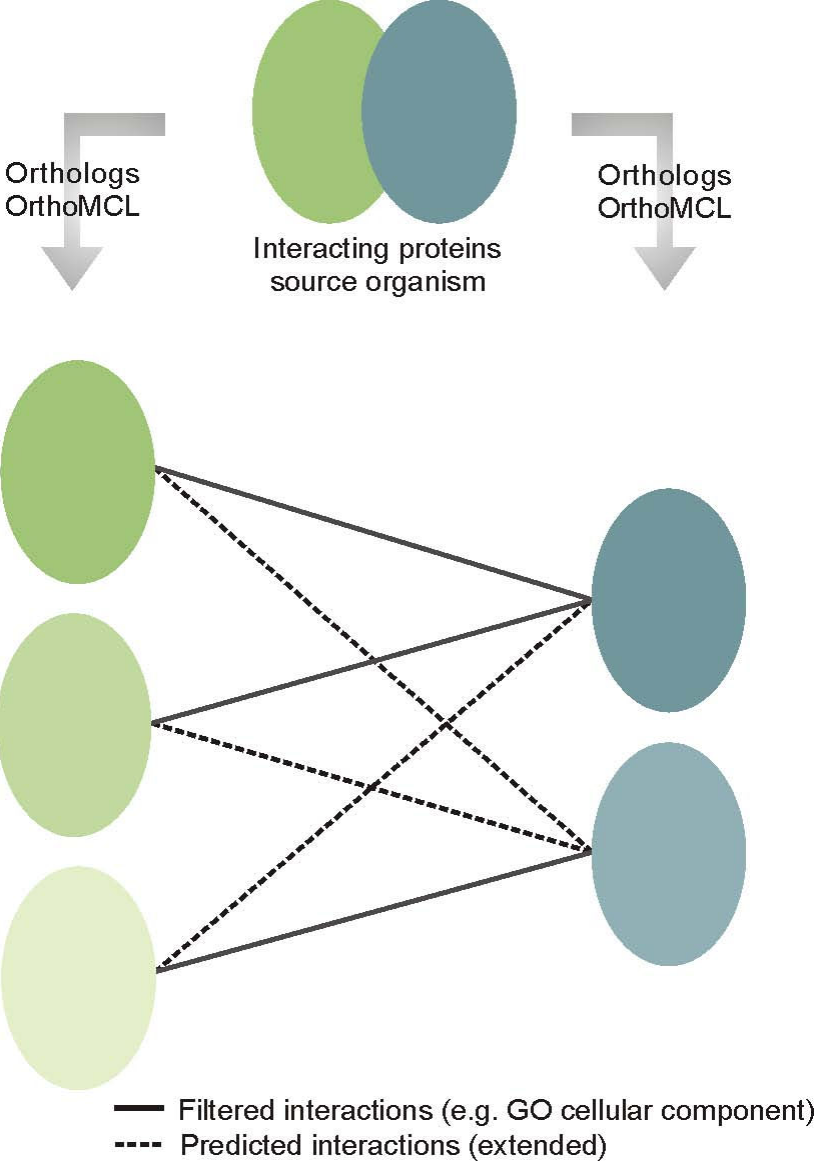
**Interolog detection and filtering**. Proteins in similar color belong to the same orthologous group as identified by OrthoMCL. Connecting lines indicate predicted interactions between proteins. First, all possible combinations between proteins in the two orthologous groups are predicted. Second, some connections between proteins do not hold true when considering the genomic features e.g. GO cellular component. The result is the filtered interactome (solid lines), which extended to the predicted interactome using the interactions that did not pass the filters (dashed lines).

The basis of the prediction of protein-protein interactions in *Arabidopsis thaliana *performed in this study resides in the detection of interologs. Interologs are defined as protein-protein interactions that are conserved between two species and that can be detected through the identification of the orthologs in the target organism of the proteins known to interact in the source organism (see Fig. 1). Applying this approach, we assume that protein-protein interactions occurring in yeast, C. elegans, Drosophila and/or human, are conserved and consequently occur in Arabidopsis as well.

**Figure 2:**
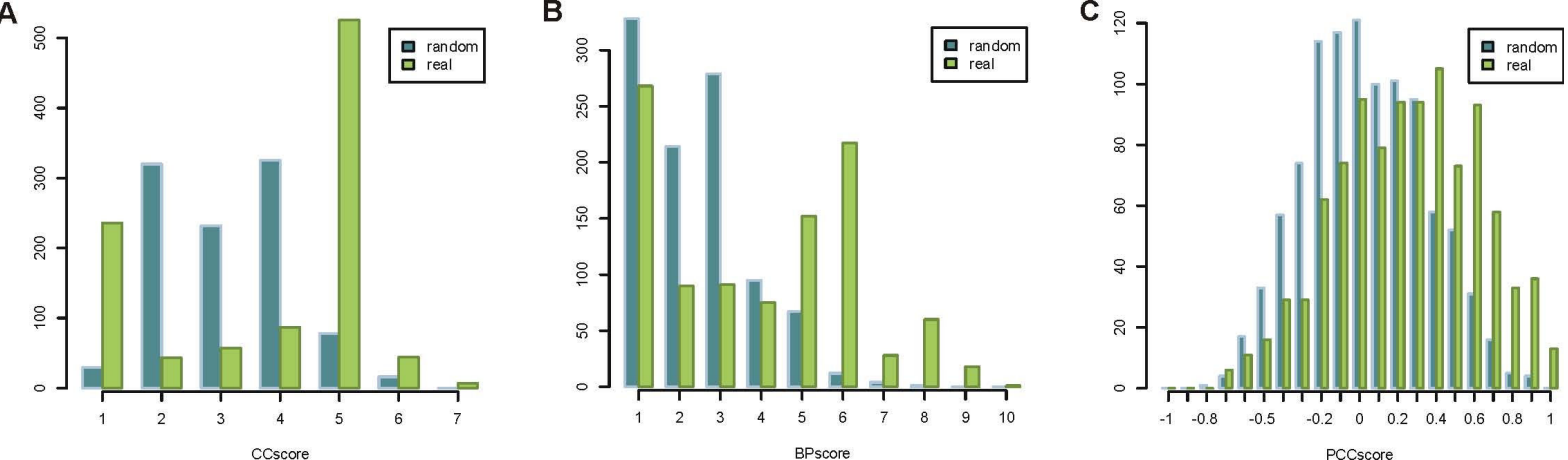
**Assessment of genomic features**. Frequency distributions of A) GO Cellular Component similarity scores, B) GO Biological Process similarity scores and C) Pearson Correlation Coefficients for real and random protein-protein interactions.

When identifying interologs, we need to deal with the high number of duplicated genes in the target organism *Arabidopsis thaliana *(see Methods; Fig. 1). Inferring all combinations of co-orthologous proteins can cause a high number of false positive predictions. To augment the confidence in the predicted interactions, we integrate data from several so-called genomic features, namely the GO biological process similarity, the GO cellular component similarity and the Pearson correlation coefficient (see Methods). Accordingly, three main conditions are set up, namely the co-localization of interacting proteins, a similar biological role for interacting proteins and the co-expression of genes encoding interacting proteins. These conditions were verified by comparing these different properties in positive and negative protein pairs (see Fig. 2). As positives, protein-protein interactions experimentally shown to interact are considered, while a negative set of protein pairs was built by making random combinations between all proteins, which is an approximation of true negative protein pairs (see Methods for details). One has to take into account that the size of the positive dataset can be relatively low and as a consequence, caution in the assessment of false positive rates needs to be taken (see Table 1; Additional file 1). For each genomic feature, the frequency distributions of positive (experimentally identified interactions) and negative (random gene pairs) datasets were compared (see Fig. 2) to choose a threshold value for the genomic features (see methods).

**Table 1:** Experimentally identified proteins and protein-protein interactions

Number of proteins	1722
Number of proteins with OG	1626

Number of proteins with a BP GO annotation (depth = 5)	1026

Number of proteins with a CC GO annotation (depth = 5)	688

Number of proteins with gene expression data	1821

Number of interactions	3035

Number of interactions with OG	3032

Number of interactions between proteins with sufficient information on different genomic features	1457

Number of interactions with a BP GO annotation (depth = 5, depth = 8)	1457,157

Number of interactions with BP score > = 5, > = 8	913, 123

Number of interactions with a CC GO annotation (depth = 5)	757

Number of interactions with CC score > = 5	565

Number of interactions with gene expression data	2482

Number of interactions with PCC> = 0.3	837

Number of interactions with a BP GO annotation (depth = 5) and expression data	1276

Number of interactions with BP score > = 5 and PCC > = 0.3)	317

Total number of filtered interactions with CC score > = 5 or BP score > = 5 and PCC > = 0.3 or BP score > = 8)	767

Number of proteins and interactions with genomic feature information for Arabidopsis protein-protein interactions from public databases (OG = orthologous group, BP = biological process, CC = cellular component, GO = gene ontology, PCC = Pearson Correlation Coefficient)

To decide if two proteins co-localize and/or function in the same biological process, both the GO cellular component (CC) and the GO biological process (BP) annotation of the interacting proteins were evaluated. To measure the similarity between the GO annotations of two proteins, we calculated the maximum depth of the common ancestor of all pairs of GO terms assigned to both proteins (see Methods; Additional file 2; Fig. 2). Similarly, the co-expression in positive and negative datasets was investigated by calculating the Pearson correlation coefficient, describing the global similarity between expression profiles for interacting and non-interacting proteins (see Fig. 2). When inspecting the distributions, a smaller difference between positive and negative datasets can be observed for expression correlation than for GO annotations. Therefore, it is impossible to define a threshold in Pearson correlation coefficient that identifies a considerable number of true positives while having few false positives (Fig. 2). Different genomic features need to be combined to maximize the coverage of the prediction (see Additional file 3). Through this combination of genomic features, thresholds may be decreased while maintaining an acceptable number of false positives (see Additional file 1 for an estimation of the false positive rates). The use of a threshold of 5 for the GO biological process similarity score in combination with a threshold of 0.3 for the Pearson correlation coefficient is acceptable (see Additional file 3; Additional file 1). Through this combination a lower threshold can be chosen for both genomic features. Nevertheless, one should keep in mind that the co-expression measure takes into account expression data from 86 microarray experiments. It can be expected that the number and type of experiments influences the overall Pearson correlation coefficients for all pairs of genes. Networks of experimentally identified protein-protein interactions in yeast and human, although containing false positive interactions as well, show an average Pearson correlation coefficient of 0.3 and 0.241 respectively, supporting our choice of the Pearson correlation coefficient threshold [36]. In addition, the GO biological process similarity score (threshold score of 8) and GO cellular component similarity score (threshold score of 5) were also used independently. These relatively high thresholds result in inferences based on very detailed GO annotations, mostly not inferred based on computational analyses (ISS)). The depth of GO terms such as ubiquitin-dependent protein catabolic process (BP ≥ 8) or peroxisome (CC ≥ 5) is high, pointing to specific processes or localizations that are mostly assigned based on experimental evidence (e.g. *inferred from direct assay*). Systematic assessment of the evidence codes of the GO terms used for the interaction filtering (BP8 and CC5) shows that a minority of terms are inferred from sequence or structural similarity (ISS) (see Additional file 4). GO terms used in the BP5+PCC0.3 filter are more often inferred from sequence or structural similarity. However, as in this case the BP score is combined with the PCC, we believe that these ISS terms do not affect the quality of our predictions considerably. Therefore, we decided to include ISS-based GO annotations throughout our analysis and only removed IPI, ND, NR, NAS and IEA-based annotations (see methods). Finally, we added up the different sets of predicted protein-protein interactions resulting from the application of different thresholds and different source organisms. We opted to add up the different sets rather than to take the intersection. On the one hand, our randomization studies show that the individual filters result in acceptable numbers of false positives. On the other hand, we encompass the relative high amount of missing functional data in cases where, for instance, BP annotation but no CC annotation is available. However, using this approach it is possible that a protein-protein interaction passed the BP8 filter, while it does not pass the CC5 filter (contradictory CC information – annotation for BP and CC available). Our systematic assessment shows that this possibility occurs for only a minority of interactions for BP8, while more often for BP5 which is used in combination with PCC0.03 (see Additional file 5). Moreover, only half of these contradictory CC annotations is inferred from experimental evidence (see Additional file 5).

In summary, 52.6% of the experimentally identified protein-protein interactions (767 out of the 1457 interactions) meet the conditions of the genomic features (GO biological process, GO cellular component, co-expression) (see Table 1 and Figs. 2 and Additional file 3), without taking into account orthology (see next paragraph).

### Prediction of protein-protein interactions in *Arabidopsis thaliana*

Starting from the protein-protein interactions in the source organisms yeast, C. elegans, Drosophila and human, protein-protein interactions were predicted in Arabidopsis using the above-mentioned genomic features. We downloaded the OrthoMCL database containing orthologous groups (OG) of 87 species. From this dataset, we extracted the evolutionary relationships between Arabidopsis, yeast, C. elegans, Drosophila and human genes. The approach taken to build this database has the advantage that in-paralogs (duplicates arisen after speciation) and thus orthologous groups rather than one-to-one orthologs can be identified. Employing these orthologous relationships between yeast, C. elegans, Drosophila and human genes on the one hand, and Arabidopsis genes on the other hand, interologs were detected (see Methods and Fig. 1 for details). The original numbers of interologs are drastically reduced when applying the genomic feature filters (see Table 2). The set of protein-protein interactions that remains after applying the genomic feature filters will further be referred to as the "filtered interactome" and accounts for 18,674 protein-protein interactions among 2233 proteins (see methods on the reliability of the predictions). However, we do not discard all interologs that do not meet the requirements of the genomic features. As we would like to study the idenfied interologs in an evolutionary context, we choose to extend the filtered interactome. For every combination of orthologous groups present in the filtered interactome, we take all other (non-filtered) interactions to build the "predicted interactome" (see Fig. 1). This predicted interactome accounts for 51,885 protein-protein interactions among 3014 proteins (see Table 2). The filtered (see Additional file 6) and predicted interactome (see Additional file 7) are provided as supplementary material at .

**Table 2:** Predicted protein-protein interactions

	**#proteins**	**#interactions**
Yeast	4662	175322

Yeast CC5	1182	9384

Yeast BP5+PCC 0.3	1249	8986

Yeast BP8	290	1336

Yeast total filtered	1919	16145

Human	3346	28397

Human CC5	694	1821

Human BP5+PCC 0.3	632	1082

Human BP8	185	421

Human total filtered	1089	2795

C. elegans	1549	4174

C. elegans CC5	149	199

C. elegans BP5+PCC 0.3	234	256

C. elegans BP8	70	100

C. elegans total filtered	330	432

Drosophila	3355	19315

Drosophila CC5	430	924

Drosophila BP5+PCC0.3	368	438

Drosophila BP8	87	140

Drosophila total filtered	646	1282

All species – Interologs with CC annotation (CC > = 5)	2123 (1431)	43698 (11891)

All species – Interologs with BP annotation (BP > = 5, BP > = 8)	3087 (2111, 416)	79475 (25037, 1890)

All species – Interologs with gene expression data (PCC > = 0.3)	5369 (4688)	161928 (62178)

All species – Filtered	2233	18674

All species – Predicted	3014	51885

All species – interologs (without filtering)	6206	216972

Number of Arabidopsis proteins and protein-protein interactions detected using different source organisms and different genomic feature filters (BP = biological process, CC = cellular component, PCC = Pearson Correlation Coefficient)

**Figure 3:**
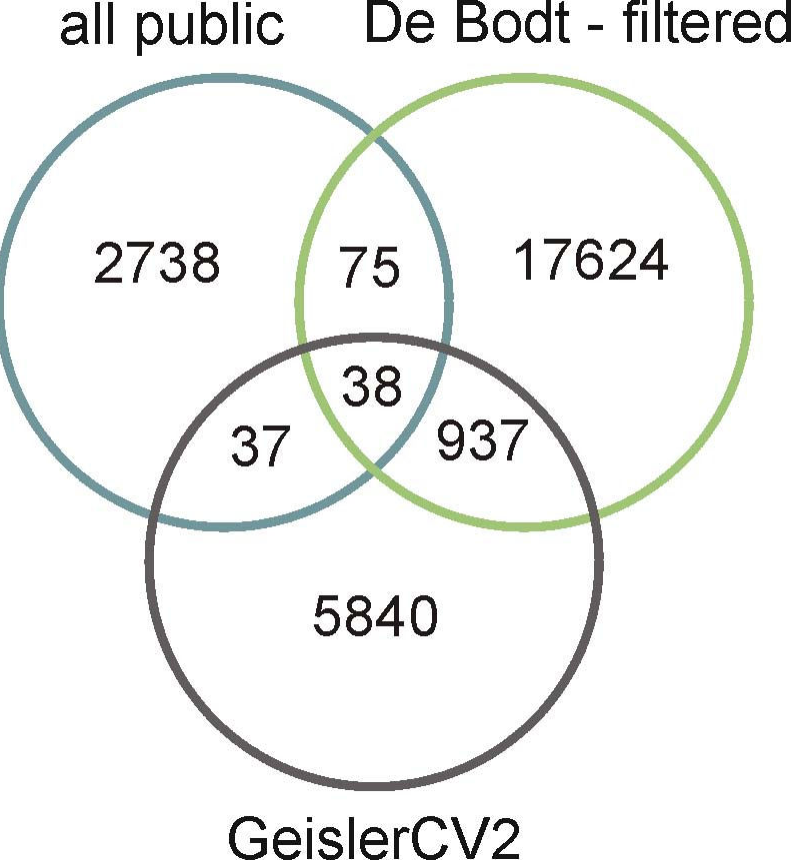
**Overlap between datasets of protein-protein interactions**. Overlap between experimentally identified protein-protein interactions available in public databases (see methods) and predicted sets from this study and the Geisler-Lee et al. study [45]. Filtered interactions from this study are compared to interactions with a confidence value of two or higher from the Geisler-Lee et al. [45] study.

The protein-protein interactions detected in the filtered and predicted interactome were compared to experimentally shown and previously predicted protein-protein interactions. Fig. 3 and Additional file 8 depict the overlap between the different datasets. Overall, a small overlap is found between our filtered interactome and the experimentally shown interactions reported by the TAIR, MINT and IntAct databases (see Fig. 3; Additional file 8, panel A1). Similarly, a small overlap of the experimentally identified interactions with the previously predicted interactions of Geisler-Lee et al. [45] and Cui et al. [46] is observed (see Fig. 3; Additional file 8, panel A2). Similar to our approach, Geisler-Lee et al. [45] identified interologs using worm, fly, human and yeast as source organisms. In this study, a confidence value is calculated taking into account the number of times a protein-protein interaction is found as interolog and/or supported by genomic features such as co-expression based on the Pearson correlation coefficient and localization based on the Arabidopsis Subcellular Database (SUBA) [47]. The predicted protein-protein interaction dataset of Cui et al. [46] was constructed based on a Naive Bayesian Classifier. This method integrates different predictive data sources such as ortholog information, GO biological process, co-expression, gene fusion, gene neighborhood, phylogenetic profiles and domain architecture, to build a model to predict novel protein-protein interactions. A comparison of our filtered and predicted interactome with these two sets of previously predicted protein-protein interactions is shown in Additional file 8 (panel B1 and B2). Although a similar approach was taken by Geisler-Lee et al. [45], a considerable number of new interactions (17624) as well as experimentally identified interactions (75) not recovered by Geisler-Lee et al. [45] is found in our study (see Fig. 3). Differences between the two approaches that may cause the relatively small overlap are most probably the use of different protein-protein interaction databases for the source organisms (BIND, MIPS, BIOGRID, and DIP were used in Geisler-Lee et al. [45]) and the use of different confidence measures. These observations corroborate previous reports on the low coverage of current protein-protein interaction datasets and detection strategies.

### Accessibility of the interactome

We have developed an easy-to-use query and visualization system to represent the inferred interactome. The discussed protein clusters as well as the complete predicted interactome can be observed through a web-start version of Cytoscape that can be found at . A node and edge attribute system is employed to represent the different types of information. The color of the edge represents the degree of co-expression calculated as the Pearson correlation coefficient (green: correlation, purple: anticorrelation), while the line width of the edge represents the GO biological process similarity score (thick: similar biological process) and the line style of the edge represents the GO cellular component similarity score (solid: similar localization). The color of the nodes corresponds to the protein cluster the protein belongs to (see further). Proteins belonging to small or no clusters are colored in grey. TAIR functional descriptions are shown as node labels. Subsets of the interactome that are of interest to the researcher can be visualized easily by querying the interactome for (a) protein(s) or a functional description.

### Delineation of protein clusters in the predicted interactome

In an attempt to reveal the functional repertoire of the predicted interactome, we have delineated highly interconnected regions in the protein-protein interaction network (hereafter called protein clusters). In addition, we tried to assign a function to the identified protein clusters. Finally, we investigated the evolutionary conservation of the protein clusters.

Identification of protein clusters is performed using the CAST clustering algorithm (see Methods). This clustering procedure employs the connectivity of the proteins. Overall, 1802 proteins taking part in 16,498 interactions could be identified in protein clusters, accounting for the majority of originally identified interactions (see Additional file 9). The biological roles of the identified protein clusters were studied through identification of overrepresented GO categories (biological process, molecular function and cellular component) (see Methods). To judge the validity of the protein clusters, we inspected clusters involved in particular biological processes together. Using this approach of clustering and subsequent GO enrichment analysis, we can elegantly pinpoint protein complexes, the relationships between them and the encompassing biological processes they are involved in (see Supplementary Data site and more details below).

**Figure 4:**
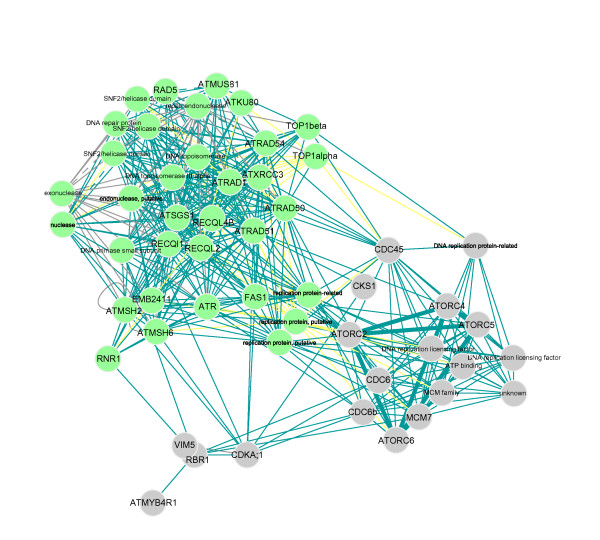
**Protein-protein interactions involved in 'DNA replication'**. Green edges represent PCC values > 0.3 (see Figure 5 for more details). For grey edges, no co-expression information is available.

As could be expected, well-conserved proteins and functions, such as those involved in transcription, translation, and proteolysis, are overrepresented. Typically, proteasome and ribosomal proteins are identified as highly connected (CAST clusters 1 and 3; see Supplementary data site). A number of protein-protein interaction networks involved in particular processes such as organelle organization and biogenesis, lipid metabolism, and ATP binding as well as the biological processes mentioned below can be viewed through our Supplementary data site. In addition, a considerable number of protein-protein interactions with a role in transmembrane transport, membrane receptor activity and vesicle trafficking were detected as previously reported by Geisler-Lee et al. [45] (see Supplementary data-Transmembrane activity). For example, a link was found between protein clusters of interacting VAMPs (Vesicle associated membrane proteins) and SNAREs (CAST clusters 10 and 70) with vacuolar H^+^ pumping ATPases (CAST clusters 9, 34 and 120) and cation/H^+^ exchangers (CAST clusters 100, 145 and 185). Although not connected to the above-mentioned clusters, a protein cluster containing components of the translocase inner membrane complex (CAST cluster 13) associated with carrier proteins (CAST cluster 94), was retrieved as well. Several links between cell cycle control, protein degradation and related processes are captured in the protein clusters enriched for GO categories containing 'cell cycle' (CAST clusters 3, 12, 18 and 61; see Supplementary data). Whereas ubiquitin-mediated proteolysis regulates the activity of cyclins during the progression through the different phases of the cell cycle, B-type cyclins interact with several microtubule-related components during cytokinesis. Similarly, A-type cyclins, DNA replication proteins (CDC, MCM and ORC subunits) and RBR/WEE proteins are associated during the G1/S transition [48] (see Supplementary data - Cell cycle + DNA repair + DNA replication). Overall, we observe a high similarity in expression patterns for the genes encoding proteins in the 'cell cycle' clusters most probably due to the tight regulation of proteins involved in DNA replication (green edges, see Fig. 4, Supplementary data). Moreover, the interactions in these clusters are often supported by the GO biological process measure (solid edges, see Supplementary data). These two properties occur mainly within protein clusters. In contrast, genes encoding interacting proteins involved in transmembrane activity, described above, show an overall lower similarity in expression patterns, even within protein clusters (yellow edges, see Supplementary data). This difference in the degree of co-expression is probably due to the more specific expression patterns of transmembrane activity genes resulting in transient interactions that cannot be identified using a global co-expression measure. However, most of the interactions in the transmembrane activity network are supported through the GO biological process and/or GO cellular component feature and thereby were identified through our approach (solid and thick edges, see Supplementary data). Reasonably, localization is very important in transmembrane activity-related processes such as ion transmembrane transport and protein excretion through vesicular exocytosis.

More detailed analysis of the 'response to' interaction network identified many proteins functioning in, for instance, response to DNA damage or response to oxidative stress caused by the accumulation of reactive oxygen species (see Supplementary data site). Proteins active in these stress responses are DNAJ heat shock proteins, calcium-dependent and MAP kinases (CAST cluster 1), DNA repair proteins (RAD1, RAD5, RAD50, RAD51, RAD54 – CAST 18), superoxide dismutases (CAST 49), glutathione peroxidases (CAST 74) and glutathione S-transferases (CAST 15). These proteins are involved in very diverse biological processes, such as response to heat, response to toxins and response to chemical stimulus, which is reflected in the dashed edges in the network (see Supplementary data). In order to verify that these predicted interactions also reflect actual conserved biological stress responses, we compared the expression patterns of all 500 Arabidopsis genes in these 'response to' protein clusters using a recently compiled comparative stress response matrix [49]. Whereas 11% of all stress-induced Arabidopsis gene families shows a conserved stress response in human or yeast (44/390 families in the complete matrix), the 'response to' set (representing 137 gene families) shows a more than five-fold enrichment for conserved stress response (16/25 gene families with responsive Arabidopsis genes are also responsive in human or yeast). These findings confirm that several components as well as protein-protein interactions in Arabidopsis indeed function in diverse responses and that this response is evolutionary conserved in eukaryotes.

Besides interactions functioning in well-conserved biological processes, we could also identify the recruitment of well-conserved proteins and protein-protein interactions in plant-specific processes. In the following examples, the regulatory mechanisms such as chromatin remodeling and actin polymerization are common to different lineages. However, these mechanisms are put into play in highly lineage-specific biological processes, such as seed and trichome development.

**Figure 5:**
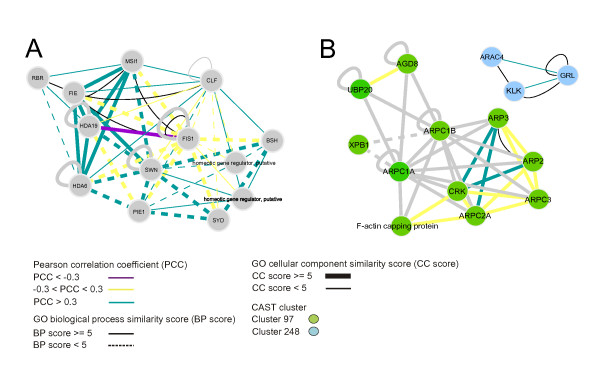
**Protein-protein interactions involved in plant-specific processes**. A. Protein-protein interactions involved in imprinting genes. B. ARP2/3 complex. Edges in black depict previous experimentally identified interactions. For grey edges, no co-expression information is available.

In a first example, we predict that the proteins FERTILIZATION INDEPENDENT ENDOSPERM (FIE), CURLY LEAF (CLF), SWINGER (SWN), MULTICOPY SUPPRESSOR OF IRA1 (MSI1), and MEDEA (MEA) interact and that this protein complex functions in flowering and embryonic development (see Fig. 5A and Supplementary data). These proteins are homologous to the Polycomb (PcG), ESC, E(Z), p55, and Su(Z)12 genes in animals and take part in the Polycomb Repressive Complex 2 (PRC2) [50]. This PRC2 complex mediates the epigenetic control of developmental patterning through chromatin modification. Genetic evidence suggests that there are different PcG complexes in plants, regulating different developmental pathways. In Arabidopsis, the FERTILIZATION INDEPENDENT SEED 2 (FIS2) repression complex is described to be involved in seed and flower development and includes FIE, MEA, MSI1, and FIS2. The VERNALIZATION2 (VRN2) complex consisting of VRN2, FIE, CLF, and SWN is involved in vernalization response. Except for VRN2, we have predicted interactions between all these components. In addition, we have uncovered additional, to our knowledge new interactors such as PHOTOPERIOD-INDEPENDENT EARLY FLOWERING 1 (PIE1), BUSHY (BSH) and SPLAYED (SYD), probable subunits of SWI/SNF chromatin remodeling complexes [51-53]. Moreover, we detect interactions with associated proteins such as histone deacytelases and putative homeotic regulators. Interesting to note is that we could also identify the interaction of MSI1 with RETINOBLASTOMA RELATED 1 (RBR1), just recently confirmed by biomolecular fluorescence complementation [54] (see Fig. 5A). This complex is overall very well supported by the genomic features used in this study (green, solid and thick edges in Fig. 5A). Moreover, several of the identified interactions were already shown experimentally (black edges in Fig. 5A).

In a second example, we have predicted interactions between different actin-related proteins (ARPs), constituting the ARP2/3 complex involved in leaf development (see Fig. 5B and Supplementary data). ARP2 or WURM, ARP3 or DISTORTED TRICHOMES 1, ARPC1, ARPC1A, ARPC2A or DISTORTED TRICHOMES 2, ARPC3 and ARPC5 or CROOKED, as well as a number of other non-actin-related proteins, were predicted to interact. The Arp2/3 complex has been shown to be composed of two actin-related proteins (Arps), a seven-bladed beta propeller (ARPC1), and four other subunits (ARPC2-5). Only ARPC4 is missing from our predictions, which is due to the fact that ARPC4 is not present in the input dataset of orthologous relationships. In addition to the ARP2/3 complex, we could identify a complex of three proteins (KLUNKER, GNARLED and ARAC4), involved in leaf development as well. At least two of these proteins of the SCAR complex are thought to regulate the ARP2/3 complex. The ARP2/3 complex functions as a central player in the precise regulation of both the initiation of actin polymerization and the organization of the resulting filaments, common to all organisms used in this study. However, disruption of the ARP2/3 complex has distinct effects in different organisms, going from growth defects in yeast, defects in eye and axon development in worm and fly, migration of cancer cells in human, and epidermal cell shape determination in Arabidopsis (Goley and Welch 2006). For the ARP2/3 protein complex, the power of the co-expression measure was limited. Nevertheless, similarity in localization and biological process showed to be very valuable. This observation points to the complementarity of the different genomic features employed in this study.

## Discussion

In this study, we have predicted protein-protein interactions in the model plant *Arabidopsis thaliana *through interolog detection with yeast, worm, fly and human as source organisms and using genomic features (expression correlation, localization and biological process) as filters to increase the confidence in our predictions. As such, a set of highly reliable interactions could be delineated that can be further employed in both computational and experimental studies. In contrast to previous efforts to predict protein-protein interactions in *Arabidopsis thaliana*, we do not only provide a list of all interologs and their associated genomic feature values, but rather focus on the subset of protein-protein interactions that is supported by the different genomic features (or the so-called filtered interactome). The extensive study of the behavior of the genomic features for experimentally identified protein-protein interactions compared to random protein pairs allowed us to validate the protein-protein interactions adequately.

The setup of this study allowed us to rigorously investigate the functional repertoire and evolutionary conservation of the identified protein-protein interactions. We conclude that although this type of protein-protein interaction prediction is highly dependent on the degree of conservation of protein-protein interactions between Arabidopsis and yeasts or animals, we were able to predict interactions with roles in diverse biological processes. Interestingly, these cover both interactions in evolutionary conserved and plant-specific processes.

Future improvements to the prediction of protein-protein interactions are manifold. For instance, it has been shown that not all protein-protein interactions are equally stable. Some interactions are permanent while others are transient, meaning that proteins only come together at certain time points or locations possibly resulting in different expression profiles of the encoding genes (just-in-time assembly) [55]. Although we could identify at least some transient interactions (e.g. interactions with transmembrane activity), the use of global expression correlation measures such as the Pearson correlation coefficient might be replaced by a measure of "partial" expression similarity that is able to capture even less stable protein-protein interactions. Recent proteomics profiling (e.g. [56]) will allow the consideration of protein activity rather than transcript activity. On the other hand, with the large-scale experimental identification of protein-protein interactions in many species, a gold standard positive set of interactions can be built more rigorously. This gold standard positive set will increase the strength of machine learning methods in protein-protein interaction detection. Nevertheless, a gold standard negative set of interactions remains problematic.

## Conclusion

In conclusion, this study showed that the integration of orthology with functional association data, such as localization, biological process and co-expression, is adequate to predict protein-protein interactions. In particular, for organisms with limited existing knowledge on protein-protein interactions, such as Arabidopsis, our approach is very valuable. On the contrary, sophisticated machine learning approaches perform poorly because of the lack of gold standard sets of interactions. We could predict a high number of new protein-protein interactions, and analysis of the functional repertoire of identified protein clusters supports the significance of these putative interactions. The approach described here can easily be adapted for estimating the reliability of experimentally identified interactions. Finally, with the growing availability of expression and gene ontology information, this approach can be applied to the detection of protein-protein interactions in agronomically and economically interesting plants, such as rice, corn and poplar.

## Methods

### Interaction data

Although numerous efforts have been made to obtain uniformity in interaction databases, interaction datasets for yeast, animals and Arabidopsis are not readily available. Protein interaction data sets were compiled from DIP [17], BioGRID [19], MINT [18] and IntAct [20] for the source organisms *Saccharomyces cerevisiae*, *Caenorhabditis elegans, Drosophila melanogaster *and *Homo sapiens *and the target organism *Arabidopsis thaliana *(for Arabidopsis, see Table 1), containing most of the large-scale interaction studies. Binary interaction data was extracted. For each interaction, the method of detection, PMID number and the bait/prey information were downloaded. As such, identical entries in the different databases were identified. In addition to the combined interaction datasets from DIP, BioGRID, MINT and IntAct, two manually curated datasets of literature-derived interactions were employed. For yeast, a set of 15,456 interactions involving 4554 proteins available at MIPS (Munich Information Center for Protein Sequences) was used. Although this curated set is half as large as the interaction dataset downloaded from the four databases, a considerable number of proteins is covered and the quality of the data is believed to be considerably higher [57]. For human, a set of 37,072 interactions involving 9565 proteins is provided by the HPRD (Human Protein Reference Database) [58]. For Arabidopsis, the experimentally identified protein-protein interactions available at TAIR  were included. Altogether, 89,537 interactions among 6515 proteins could be found in public databases of *Saccharomyces cerevisiae*, 8167 interactions among 4126 proteins of *Caenorhabditis elegans*, 56,088 interactions among 14,112 proteins of *Drosophila melanogaster*, 60,775 interactions among 15,126 proteins of *Homo sapiens*, and 3587 interactions among 1722 proteins of *Arabidopsis thaliana *(see Table 1).

Negative datasets were built by randomizing protein pairs. To analyze equal sample sizes and to take into account the availability of genomic feature data, the negative as well as the positive datasets contained 1000 protein pairs for the assessment of individual genomic features and 500 protein pairs for the combined genomic features. This approach has the disadvantage that some positive pairs may be included in the dataset. However, the number of positive pairs will be extremely low taking into account the number of possible combinations between all Arabidopsis proteins and the estimated size of interactomes of higher organisms. An alternative approach would be to consider pairs that consist of proteins that are not present in the same cellular compartment. However, this method can be biased as not all proteins that localize in the same cellular compartment interact. Moreover, in this study, we use the cellular component annotation to identify positive interactions. Consequently, we opted for the randomization approach throughout our study.

Positive and negative datasets were compared to choose appropriate thresholds for the genomic features (similarity in expression, biological process and cellular localization, see further). We have compared balanced datasets (equal number of positive and negative interactions) to estimate the reliability of our genomic feature filtering. The thresholds chosen in this study would correspond to a positive predictive value (number of true positives/(number of true positives + number of false positives)) of 95% in a one to one ratio. However, in reality, although difficult to estimate, the positive and negative protein pairs do not occur in a one to one ratio. Therefore, the positive predictive value is actually smaller. In a one to ten or one to 100 ratio, the PPV drops to ~50% or ~30%, respectively. However, these positive predicted values are probably not robust and should be considered with caution due to the small sample size (sparse distributions of genomic features of the positive dataset compared to the negative dataset). The number of positive interactions for which the genomic features pass the thresholds is extremely low, probably due to the fact that so few protein-protein interactions have been experimentally identified and/or possess sufficient gene ontology information. Even more importantly, the calculation of these positive predictive values does not take into account the fact that, through the interolog detection, our initial predictions (before application of genomic feature thresholds) are already enriched for true interactions. Through this step, the PPV increases to 88% and only the most likely interactions remain.

### Identification of interologs

Whereas earlier methods used BLAST to identify orthologous genes, nowadays more dedicated tools such as OrthoMCL and INPARANOID, which take into account in-paralogs (duplicates arisen after speciation), are applied [59-61]. In this study, orthologous relationships were identified based on the OrthoMCL database containing orthologous groups for 87 species [61]. Like INPARANOID [59], the OrthoMCL software takes into account in-paralogs (genes duplicated in one species after speciation with another species) [62]. We extracted data from five organisms, namely the source organisms *Saccharomyces cerevisiae, Caenorabditis elegans, Drosophila melanogaster *and *Homo sapiens*, and the target organism *Arabidopsis thaliana*. For a certain pair of interacting proteins in the source organism, all combinations between the (co-)orthologous proteins in the target organism were made (see Fig. 1). From all combinations only reliable interactions are identified using the genomic feature filters described below. As the genomic feature filters can not be used to assess the reliability of self-interactions (homodimers), these interactions are excluded from the filtered interactome.

### Gene ontology information

The Gene Ontology (GO) consortium provides a structured standard vocabulary for describing the function of gene products [63]. It is divided into three ontologies: biological process, molecular function and cellular component, represented by directed acyclic graphs in which nodes correspond to GO terms and edges to their relationships. For each protein, GO terms (GO cellular component and biological process annotation) were extracted from the Gene Ontology database [64] and annotations for Arabidopsis proteins were downloaded from TAIR [65]. These GO terms were used to assess the relatedness of interacting proteins by calculating a GO similarity score (see Additional file 2). We test if interacting proteins are localized in the same cellular compartment and if interacting proteins function in the same biological process. For each protein pair, all GO terms of both proteins are compared to each other. For each pair of GO terms, the depth of the common ancestor of the terms, which is the shortest path of the common ancestor to the root (GO:0003673), is calculated. Subsequently, the maximum value of the calculated depths is taken as the GO similarity score for a certain protein pair. Although this disregards how far away the GO terms are from their common ancestor, this approach has proven valuable for the aims put forward in this study, namely distinguishing between actual protein-protein interactions and random protein-protein pairs (see Results).

For both cellular component and biological process annotation, GO term assignments based on physical interactions (IPI, see  for details on evidence codes) or electronically assigned and less reliably assigned GO terms (with evidence codes ND, NR, NAS and IEA) are removed. Although the number of proteins with a GO annotation decreases considerably, the reliability of the GO similarity scores increases through this procedure (data not shown). Nevertheless, ISS-based annotations were included. We rigorously assessed the possibility of including annotations based on ISS as this accounts for a considerably number of annotations and could conclude that the low reliability of these annotations does not pose a problem to our approach (see Additional file 4; Additional file 5; see Results).

### Gene expression data

A heterogenic set of microarray expression data containing amongst others growth, stress, and mutation experiments (86) was compiled from NASC (Nottingham Arabidopsis Stock Centre) to detect co-expression between Arabidopsis genes [66]. Microarray experiments with at least 2 replicates were taken. Expression values were processed using RMA (robust multichip average) [67,68]. Co-expression was identified through the calculation of Pearson correlation coefficients (PCC) between the expression profiles of genes possibly encoding interacting proteins.

### Clustering of protein-protein interactions

The predicted interactome can be represented as a graph of nodes, corresponding to the proteins, and connecting edges, corresponding to the interactions. Protein complexes were delineated from this graph making use of the Cluster Affinity Search Technique (CAST) algorithm [69]. This algorithm was originally designed to identify clusters of co-expressed genes. For this purpose, a measure for co-expression of two genes, e.g. the Pearson correlation coefficient, is used as the weight of an edge. However, in this study, all edges were treated equally, avoiding a bias towards protein-protein interactions for which the encoding genes have highly similar expression profiles. A cluster is initiated by choosing the protein with the maximum number of neighbors using a heuristic independent from the CAST algorithm. Subsequently, neighbors of that protein are added to the protein cluster if the neighbor is connected to more than 25% of the proteins already present in the cluster. Although the connectivity of the protein clusters depends on the functional role of the cluster, we could conclude that a connectivity of 25% (compared to 0%, 50%, 75% and 100%) yielded the most robust and functionally relevant protein clusters (data not shown).

### GO overrepresentation analysis

The identified protein complexes are subjected to functional analysis. The assignments of genes to the original GO categories were extended to include parental terms (that is, a gene assigned to a given category was automatically assigned to all the parent categories as well). All GO categories containing less than 20 genes were discarded for further analysis. Enrichment values were calculated as the ratio of the relative occurrence in a set of genes to the relative occurrence in the genome. Overrepresentation of GO categories (biological process, molecular function and cellular component) was tested using the Fisher exact test. P values were adjusted using the Bonferroni correction for multiple hypotheses testing. GO categories are assumed to be significantly overrepresented when the corrected P value is smaller than 0.01.

## Authors' contributions

SB and PR conceived the study. SB and KV designed the study. SP was responsible for data retrieval and predictions. SB performed the assessment of genomic features, predictions and biological interpretation. KV performed clustering and GO overrepresentation analysis of the predictions. SB and KV drafted the manuscript. YVDP and PR critically revised the manuscript. All authors read and approved the final manuscript.

## Supplementary Material

Additional file 1 **False positive rates of different combinations of genomic features**. BP = biological process, CC = cellular component, PCC = Pearson correlation coefficient. Click here for file

Additional file 2 **Calculation of GO similarity score**. All possible GO terms of two proteins are compared in a pairwise manner. For each pair of GO terms (in green), the depth of the common ancestor (in blue) of these terms is calculated. The maximum depth of all pairwise combinations of GO terms is considered as the GO similarity score between two proteins. Click here for file

Additional file 3 **Figure S2**. Assessment of combinations of genomic features. Click here for file

Additional file 4 **Table S2**. Assessment of GO evidence codes. Click here for file

Additional file 5 **Table S3**. Possible contradictory annotations. Click here for file

Additional file 6 **Filtered interactome**. Protein-protein interactions that passed the genomic feature filters. BP score > = 8, CC score > = 5 or BP score > = 5 + PCC > = 0.3. Click here for file

Additional file 7 **Predicted interactome**. Extension of filtered interactome. Interactions with orthologous group combination already present in filtered interactome are included. Click here for file

Additional file 8 **Overlap of the different sets of protein-protein interactions**. Overlap of the filtered (1) and predicted (2) interactome with previously experimentally shown (A) and predicted protein-protein interactions (B). Click here for file

Additional file 9 **CAST clusters and GO overrepresentation**. Click here for file
