# Supplementary material for: Predicting protein-protein interactions in Arabidopsis thaliana through integration of orthology, gene ontology and co-expression
Source: BMC Genomics. 2009 Jun 29;10:288. doi: 10.1186/1471-2164-10-288 (PMC2719670; doi:10.1186/1471-2164-10-288)
Supplement: Additional file 1 — False positive rates of different combinations of genomic features. BP = biological process, CC = cellular component, PCC = Pearson correlation coefficient. [file 1471-2164-10-288-S1.doc]

**Table S1.** False positive rates for different combinations of genomic features (BP=biological process, CC=cellular component, PCC=Pearson Correlation Coefficient)

| **Genomic feature** | **False positive rate** |
| --- | --- |
| BP4 + PCC0.3 | 9/169=0.053 |
| **BP5 + PCC0.3** | **6/142=0.042** |
| CC5 + PCC0.3 | 2/167=0.012 |
| BP5 + CC5 + PCC0.3 | 2/130=0.0154 |
| BP4 + CC5 + PCC0.3 | 2/144=0.0138 |
| BP5 + CC5 + PCC0.2 | 2/150=0.0133 |
| BP4 + CC5 + PCC0.2 | 3/165=0.0182 |
| PCC0.2 | 154/253=0.609 |
| PCC0.3 | 113/213=0531 |
| PCC0.5 | 59/121=0.488 |
| BP5 | 28/284=0.099 |
| **CC5** | **12/384=0.031** |
| BP6 | 19/225=0.084 |
| BP7 | 10/177=0.056 |
| **BP8** | **2/104=0.019** |
