# Supplementary figures and images for: Predicting protein-protein interactions in Arabidopsis thaliana through integration of orthology, gene ontology and co-expression
Source: BMC Genomics. 2009 Jun 29;10:288. doi: 10.1186/1471-2164-10-288 (PMC2719670; doi:10.1186/1471-2164-10-288)

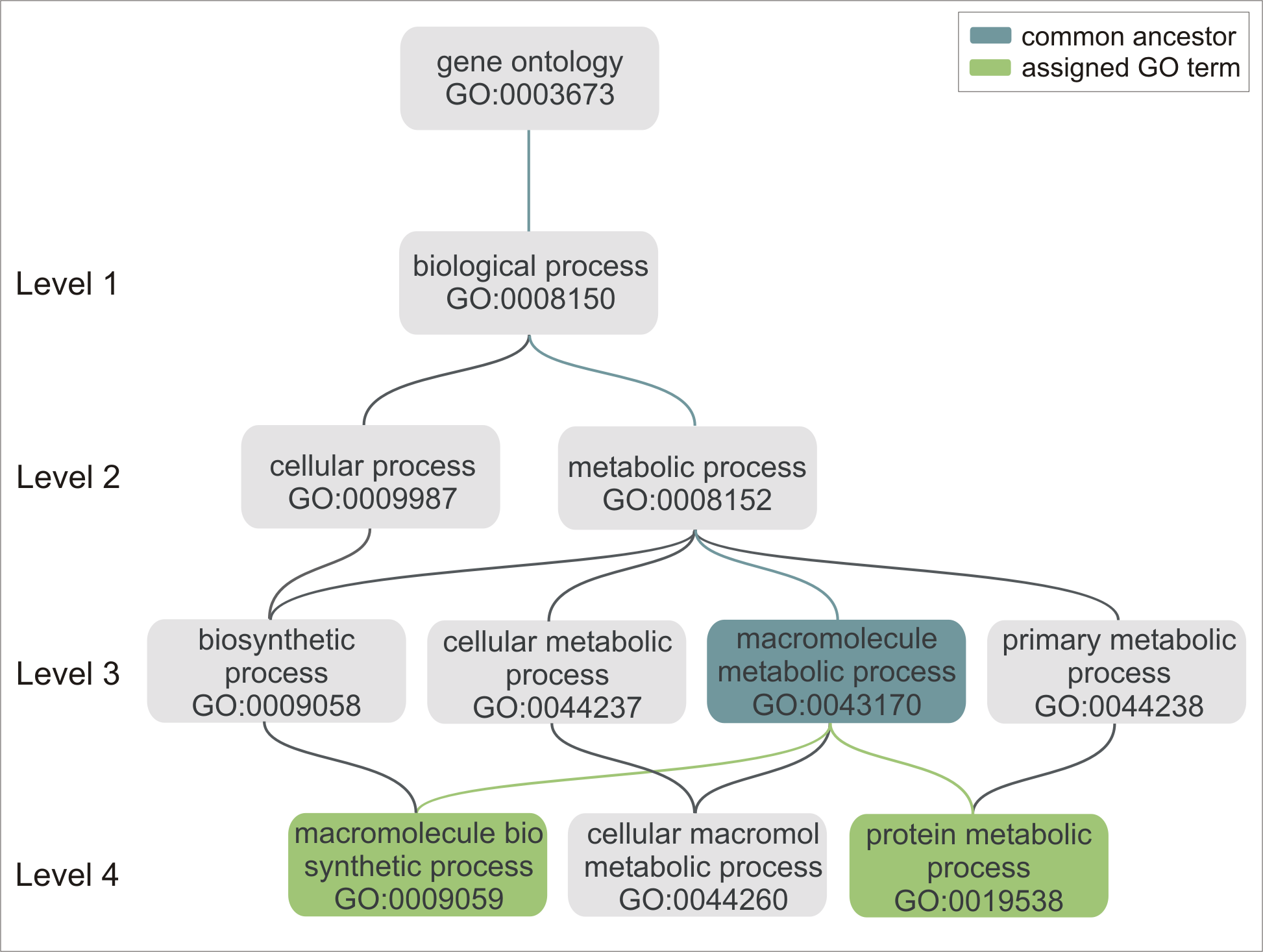

Supplement: Additional file 2 — Calculation of GO similarity score. All possible GO terms of two proteins are compared in a pairwise manner. For each pair of GO terms (in green), the depth of the common ancestor (in blue) of these terms is calculated. The maximum depth of all pairwise combinations of GO terms is considered as the GO similarity score between two proteins. [file 1471-2164-10-288-S2.tiff]

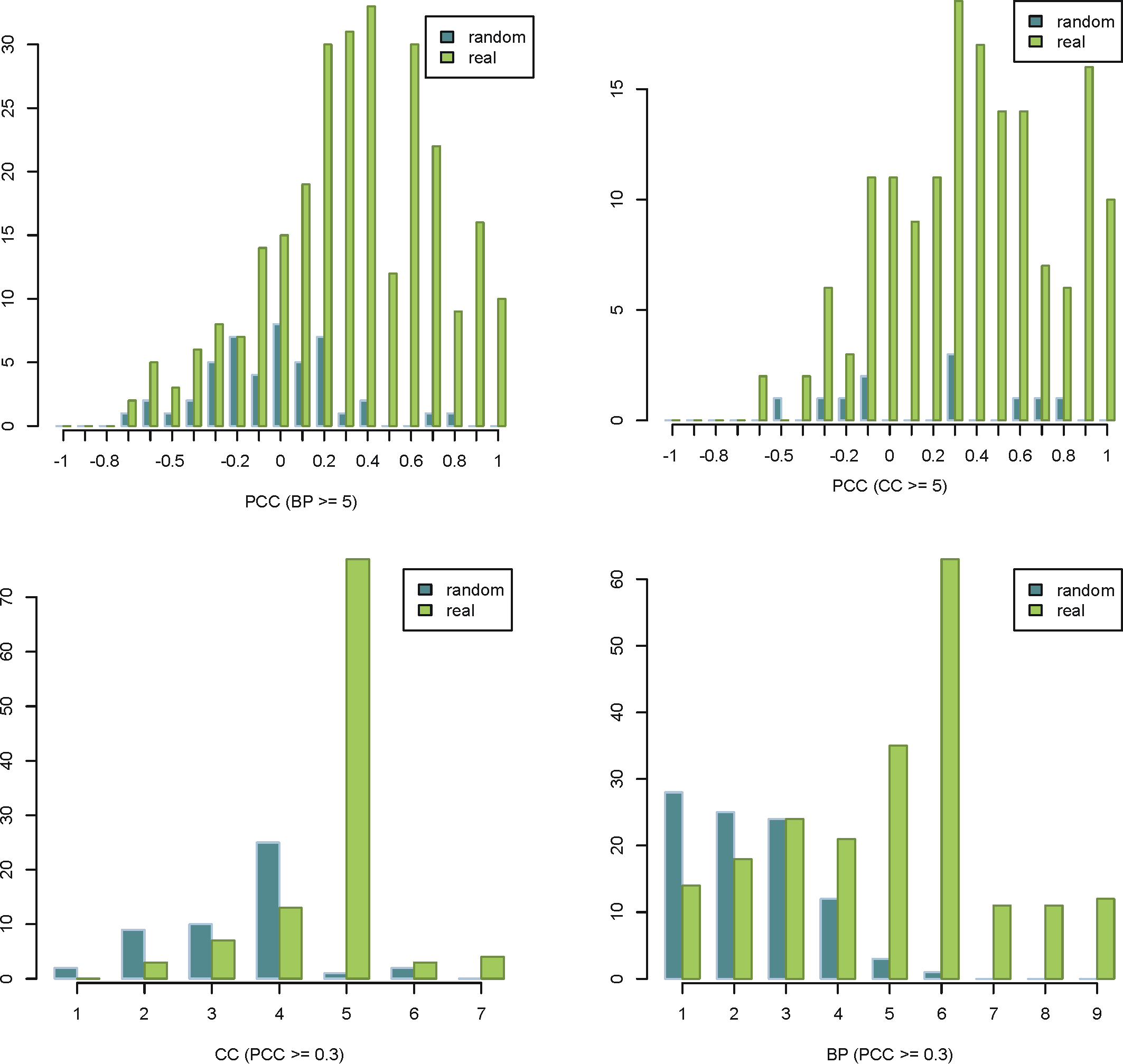

Supplement: Additional file 3 — Figure S2. Assessment of combinations of genomic features. [file 1471-2164-10-288-S3.tiff]
